# Technological and Sensory Quality and Microbiological Safety of RIR Chicken Breast Meat Marinated with Fermented Milk Products

**DOI:** 10.3390/ani11113282

**Published:** 2021-11-16

**Authors:** Zofia Sokołowicz, Anna Augustyńska-Prejsnar, Józefa Krawczyk, Miroslava Kačániová, Maciej Kluz, Paweł Hanus, Jadwiga Topczewska

**Affiliations:** 1 Department of Animal Production and Poultry Products Evaluation, Institute of Food and Nutrition Technology, University of Rzeszow, 35-959 Rzeszow, Poland; zosokolo@ur.edu.pl (Z.S.); jtopczewska@ur.edu.pl (J.T.); 2 Department of Poultry Breeding, National Research Institute of Animal Production, Krakowska Street 1, 32-083 Kraków, Poland; jozefa.krawczyk@iz.edu.pl; 3 Department of Bioenergetics, Food Analysis and Microbiology, Institute of Food and Nutrition Technology, University of Rzeszow, 35-959 Rzeszow, Poland; kacaniova.miroslava@gmail.com (M.K.); mkluz@univ.rzeszow.pl (M.K.); 4 Institute of Horticulture, Faculty of Horticulture and Landscape Engineering, Slovak University of Agriculture, 949 76 Nitra, Slovakia; 5 Department of Food Technology and Human Nutrition, Institute of Food and Nutrition Technology, University of Rzeszow, 35-959 Rzeszow, Poland; phanus@ur.edu.pl

**Keywords:** old layer hen meat, microbiological quality, sensory quality, buttermilk, sour milk

## Abstract

**Simple Summary:**

The use of meat from hens after the end of the laying period is limited due to their inferior sensory properties compared to the meat of young slaughter birds, mainly due to the age of the hens. Therefore, we are looking for effective methods of softening the meat of laying hens after the end of the annual laying use. One way to reduce the hardness of hen meat after the laying period is to marinate it with fermented milk products. The aim of the research was to evaluate the effect of marinating with buttermilk and sour milk on the quality of Rhode Island Red (RIR) hen meat after the first year of laying use. In the conducted research, it was found that marinating hen meat after the first year of laying with fermented milk products has a beneficial effect on the characteristics of raw and roasted meat. Roasted hen meat was characterised by a brighter colour, lower hardness, and better microbiological quality, and had greater overall acceptability. The obtained results allow us to conclude that marinating hen meat with fermented milk products creates new opportunities and prospects for the culinary use of the meat of RIR hens after one year of laying use.

**Abstract:**

The aim of the study was to determine the effect of marinating with fermented milk products (buttermilk and sour milk) on the physical characteristics, microbiological quality, and sensory acceptability of Rhode Island Red (RIR) hen meat after the first year of laying use. The hen breast meat was marinated with fermented dairy products, buttermilk and sour milk, by the immersion method for 12 h at 4 °C. The assessed features included the quality of raw and roasted marinated and non-marinated meat in terms of physical characteristics (marinade absorption, water absorption, pH, L*, a*, b* colour, shear strength, texture profile analysis (TPA) test), microbiological parameters, and sensory characteristics. Bacteria were identified by the mass spectrometry method (MALDI-TOF MS Biotyper). Marinating meat with fermented dairy products lightened the colour, decreased the value of shear force, reduced hardness and chewiness, and limited the growth of aerobic bacteria and *Pseudomonas* spp. Additionally, after heat treatment, the number of identified aerobic bacteria families in the marinated in buttermilk and marinated in sour milk groups was smaller than in the non-marinated muscle group. The sensory evaluation showed a beneficial effect of marinating with buttermilk and sour milk on the tenderness, juiciness, and colour of roasted meat.

## 1. Introduction

The production of poultry meat is based on the rearing of young slaughter birds (broiler chickens). The use of meat from commercial hens after the end of the laying period is limited due to their inferior sensory properties (mainly hardness) compared to the meat of young slaughter birds, mainly due to the age of the hens [1]. The development of intensive poultry production has minimised the importance of the two-way use of hens and the use of native and local breeds. An example of hens that in the first half of the twentieth century in Poland were used in two directions, i.e., for the production of eggs and meat, and in commercial production have been replaced by high-productivity hybrids intended for the intensive production of eggs or poultry meat are Rhode Island Red (RIR) hens. Due to the development of the one-way use of hens and the drastically declining population in Poland in 2003, RIR (R-11) hens were included in the program for the protection of genetic resources of poultry. Rhode Island Red (R-11) hens are typical representatives of general utility breeds. They are characterised by a large body weight, which is 2200–2600 g in laying hens [2]. RIR hens are particularly suitable for breeding in systems with free access to green run due to their adaptations to local environmental conditions. Currently, RIR hens are most often used in organic or free-range breeding conditions, and after the end of the laying period, their carcasses are most often used to prepare broths. The increase in demand for local and regional food products observed in recent years creates new opportunities and prospects for the use of RIR hens, not only for the production of eggs but also for the use of their meat after the laying period. In the opinion of many consumers, the meat of R-11 hens from free-range breeding, as opposed to the meat of laying hens from commercial breeding, after the end of the first year of laying, due to its excellent taste, may be an attractive culinary raw material for the preparation of local and regional dishes [3].

Microbiological safety is a very important problem in the modern economy and food production. Food of poultry origin is an issue of special concern, as poultry meat is characterised by a higher level of bacterial contamination than beef or pork. Poultry meat belongs to perishable food products because it makes a good substrate for the development of microorganisms, both those that cause the spoilage of meat and pathogenic microorganisms. The microbiological quality of the final product depends mainly on the microbiological quality of raw meat. The bacteria in the meat can come from the birds themselves as well as from their habitat. The presence and abundance of microorganisms may also be influenced by processing conditions and the use of various additives in the processing [4]. Thanks to the development of modern methods of assessing the microbiological quality of meat and the use of the mass spectrometry method (MALDI-TOF MS Biotyper), it has become possible to quickly assess the microbiological quality of poultry meat and to identify bacteria defined as indicators of hygiene or the deterioration of meat [5], such as *E. coli*, or *Pseudomonas* spp. [4,6]. The processing and the ingredients used may have a negative effect on the meat microbiota, causing a negative impact and accelerating the spoilage of the meat product, or may have a positive effect on the product quality and safety. One of the possibilities of improving microbiological safety and extending the shelf life of meat may be the use of natural marinades, which at the same time improve the sensory properties of meat. 

In the opinion of consumers, tenderness is one of the most important attributes of the food quality of meat [7] and depends both on the histochemical properties of the muscles [8], their structure, and the properties of the myofibrils and connective tissue that surround the muscle fibres [9]. Marinating may be a way to improve the texture characteristics of meat, including tenderness. Marinating usually involves immersing the meat, usually in an acidic solution with the addition of spices or herbs, to give the product a specific taste, enhance sensory values, and soften the meat [10,11]. The study by Gök and Ybor [12] showed that by marinating poultry meat with fruit juice marinade, a product with better taste qualities and longer shelf life can be obtained. In the authors’ opinion, it would be beneficial to use local fermented milk products to develop a recipe for new local products based on RIR hen meat after one year of laying use. The use of fermented milk products as the main ingredient in marinades for pickling poultry meat has not received sufficient attention in the scientific community, probably because the meat of young slaughter birds (broiler chickens), which is the basis for the production of poultry meat, has good texture characteristics and does not need to be treated to be softened [13]. The meat of old hens after laying, due to its high hardness, does not meet the expectations of consumers and requires softening, e.g., by marinating. The use of marinades with fermented dairy products may have a beneficial effect on the quality and microbiological safety of meat products, as well as sensory acceptability.

The aim of the research was to determine the effect of marinating with fermented milk products on the physical characteristics, as well as microbiological quality and sensory acceptability, of RIR hen meat after one year of laying use.

## 2. Materials and Methods

### 2.1. Research Material

The study included breast muscles obtained from Rhode Island Red (RIR) hens. Hens were housed in a poultry house in the deep litter with free access to a grass-covered open-air run. Indoor stocking density was 6 hens/m^2^, while outdoor stocking density was one laying hen per 4 m^2^. The hens were slaughtered after the end of the first year of laying, i.e., at the age of 64 weeks. Twenty-four hours after slaughter, the skinless breast muscles were manually trimmed from the chilled carcasses [14]. The obtained individual breast muscles were randomly assigned to three groups. The first control group (C) contained breast muscles (n = 60) that had not been marinated, the second group (n = 60) breast muscles that were marinated with buttermilk (MB), and the third group (n = 60) breast muscles that were marinated with sour milk (MS). Before marinating, all breast muscles were individually weighed with an accuracy of 0.01 g and marked with a three-digit code. 

### 2.2. Marinating Process

Marinating was carried out by the immersion method for 12 h at 4 °C, in plastic containers approved for contact with food. In the marinating process, the breast meat of the BM hens was dipped in buttermilk and the breast meat of the MS hens was in sour milk. In each group, the ratio between the weight of the meat (g) and the marinade volume (mL) was 1:2. This amount of marinade was sufficient to completely immerse the muscles in the marinade. Non-marinated muscles (NM) were stored under the same thermal conditions as marinated muscles until evaluation. After marinating, the breast meat was allowed to drain for 5 min and weighed again. 

Fermented dairy products used for marinating, i.e., buttermilk and sour milk were produced by a local producer of organic fermented dairy products. These products are on the retail market and available to consumers. The buttermilk used for marinating breast muscles from the BM group was a fermented milk product made from normalised milk with a content of 1.5% fat, concentrated by adding milk proteins, subjected to the pasteurisation process, and then acidified with cultures of lactic acid bacteria: *Lactococcus lactis* subsp. *cremoris*, *Lactococcus lactis* subsp. *lactis*, *Lactococcus mesenteroides* subsp. *cremoris*, *Leuconostoc pseudomesenteroides*, and *Lactococcus lactis* subsp. *lactis biovar diacetylactis*. Sour milk is a popular dairy product made by acidifying milk using various lactic acid bacteria (LAB). In the present study, the sour milk used to marinate meat from the SM group was made using the thermostat method from pasteurised milk with a fat content of 1.5%, acidified with bacterial cultures: *Lactococcus lactis* subsp. *cremoris*, *Lactococcus lactis* subsp. *lactis biovar diacetylactis*, *Lactococcus lactis* subsp. *lactis*, and *Leuconostoc* spp.

Before marinating, the chemical composition of buttermilk and sour milk was analysed. Each test was performed in triplicate using a Bentley B-150 Milk and Milk Product Chemical Composition Analyzer (Bentley, Chaska, Minnesota, MN, USA). The buttermilk contained 3.8% protein, 1.5% fat, and 5.1% carbohydrates and its energy value was 207 kJ (49 kcal). Sour milk with an energy value of 210 kJ/50 kcal contained 3.9% protein, 1.5% fat, and 5% carbohydrates. 

### 2.3. Samples Cooking

Samples of non-marinated (C) and marinated (BM and SM) breast muscles were cooked in a 180 °C oven to a final temperature of 80 °C at the centre of the muscle sample. Internal temperatures were monitored with a digital thermometer with an external probe. Samples were removed from the oven and tempered for 5 min before weighing each individual piece. The test samples were weighed before and after the roasting process with an accuracy of 0.01 g (Ohaus V1193, Parsippany, NJ, USA).

### 2.4. Evaluation of the Physical Characteristics of Meat

To determine the marinade absorption, meat samples were weighed before and after marinating. The marinade absorption was calculated as: Marinade absorption (%) = Weight of sample after marinating (g) − Weight of sample before marinating (g) × 100 /Weight of sample before marinating (g). The pH values of the breast muscle were measured at 45 min post-mortem at 1 cm depth using a portable pH meter (HI 99163 Hanna Instrument Company, Vöhringen, Germany). Before analysis, the meter was adjusted using buffer solutions (Hanna Instrument, Salaj, Romania) with pH values of 4.01 and 7.01 at room temperature. The average pH value was defined from three measured values of the same area, and the procedures were the same for all of the samples. The meat colour of the breast muscle was evaluated using a colourimeter (CR-300; Minolta Camera, Osaka, Japan), which was calibrated according to the manufacturer’s manual before analysis. Colour was evaluated immediately after the samples were removed from the marinades. The tests were performed on the surface and freshly cut cross-sectional area of the samples along the muscle fibres. Three measurements were made for each test, and the final value for each sample was the average of these readings. Meat colour was shown as lightness (L*), redness (a*), and yellowness (b*) in accordance with the International Commission on Illumination (CIE) colour systems. Hardness was measured based on the cutting force (Fmax), using a Zwick/Roelltesting machine BT1-FR1.OTH.D14 (from ZwickCmbH& Co.KG., Ulm, Germany), applying a wide-width Warner–Bratzler (V-blade) with a head speed of 100 mm·min^−1^ and a 0.2 N pre-cut force. The cutting was carried out on meat cubes with a cross-section of 100 mm^2^ and a length of 50 mm. Texture profile analysis (TPA) was performed using a Texture Analyser CT3 25 (Brookfield, Middleboro, MA, USA) equipped with a cylindrical probe with a diameter of 38.1 mm and a length of 20 mm. The texture was determined in samples with dimensions of 20 mm × 20 mm × 20 mm. A test of the double compression of the samples to 50% of their height was made. The speed of the roller movement during the test was 2 m/s, and the gap between pressures was 2 s. The TPA parameters: hardness (N; peak force during the first compression), springiness (mm; speed of the test sample returning from the deformed state to the initial state), cohesiveness (strength of internal bonds forming the product framework), gumminess (N; hardness × cohesiveness), and chewiness (mJ; gumminess × springiness) were calculated from the force–time curves recorded for each sample using Texture Pro Weight loss (%) was calculated using the formula weight before roasting-weight after roasting/weight before roasting × 100.

### 2.5. Microbiological Analysis

The material was collected from hen breast muscles (10 g) using sterile instruments. The samples were placed in a sterile stomacher bag. The samples were homogenised from 90 mL of 0.1% peptone water with pH = 7.0 for 30 min at 20 °C. Serial dilutions were made from 10^−1^ to 10^−3^. Samples were cultured on Trypticasein Soy Lab-Agar (TSA, Biocorp, Cournon-d’Auvergne, France) to determine the total number of mesophilic aerobic microorganisms to calculate the parameters of colony-forming units per gram of sample (cfu/g), and samples were incubated for 24 h at 37 °C under aerobic conditions. In the case of *Pseudomonas* spp., a medium for the isolation of *Pseudomonas* (PIA, Oxoid, Hampshire, UK) was used, and the samples were incubated for 48 h at 25 °C under aerobic conditions. Violet Red Bile Lactose Agar (VRBL, Biocorp, Cournon-d’Auvergne, France) was used to isolate Enterobacteriaceae. The inoculated plates were incubated at 37 °C for 24 h. The test was as follows, performed in 3 repetitions. Samples for microbiological evaluation after roasting were taken after 24 h of storage in a cold store (FKv 36110, Liebherr, Donau, Germany) at 4 °C ± 1 °C.

### 2.6. Mass Spectrometry Identification of Isolates

The sample for MALDI-TOF MS Biotyper analysis was prepared according to the extraction procedure provided by the manufacturer (Bruker Daltonik, Bremen, Germany). The bacterial colony was suspended in 300 μL water (Sigma-Aldrich, St. Louis, MO, USA) and 900 μL absolute ethanol (Bruker Daltonik, Bremen, Germany), ten times mixed and centrifuged at 13,000 rpm for 2 min. The supernatant was rejected, and the pellets were centrifuged several times. After removal of the supernatant, the pellets were mixed with 10 μL 70% formic acid (*v*/*v*) (Sigma-Aldrich, Saint Louis, MO, USA) and the same volume of acetonitrile (Sigma-Aldrich, Saint Louis, MO, USA). The mixture was repeatedly centrifuged and stained with 1 μL of the supernatant on a polished steel target plate and air-dried at room temperature. To each sample was applied 1 μL of MALDI matrix (saturated solution of α-cyano-4-hydroxycinnamic acid, HCCA, Bruker Daltonik, Bremen, Germany) in 50% acetonitrile and 2.5% trifluoroacetic acid (Sigma-Aldrich, Saint Louis, MO, USA). The mass spectrometry results were generated automatically by the Microflex LT MALDI-TOF mass spectrometer (Bruker Daltonik, Bremen, Germany) working in a linearly positive mode in the mass range 2000–20,000 Da. The device was calibrated using the Bruker bacterial standard. Spectrometric results were processed using MALDI Biotyper 3.0 software (Bruker Daltonik, Bremen, Germany). The following identification criteria were used: a score of 2300 to 3000 indicated highly probable identification at the species level; a score of 2000 to 2299 indicated safe genus identification with probable species identification; and a score of 1700 to 1999 indicated probable identification at the genus level.

### 2.7. Sensory Evaluation

Evaluation of the sensory characteristics of marinated and non-marinated hen breast meat samples after thermal treatment was carried out using the scaling method, according to a 5-point scale [15]. The selection of people for the evaluation team, as well as the training to check the sensory sensitivity of the candidates for the sensory evaluation team, was conducted in accordance with ISO [16,17] standards. The evaluation team consisted of 9 people with confirmed sensory sensitivity and with at least 3 years of experience in the field of sensory evaluation. For evaluation, after roasting, the meat was cooled to room temperature and cut into 1 cm × 1 cm × 3 cm pieces. The individual samples intended for evaluation were placed in plastic containers with a lid and marked with a number–letter code. The conditions in the room (temperature 20 °C, lighting of individual evaluation stands) ensured the comfort of work for the evaluators and allowed for an independent evaluation. Between consecutive tests of meat samples, members of the evaluation team rinsed their mouths with water. 

### 2.8. Statistical Analysis

The obtained data were collated and submitted for statistical analysis using Statistica 13.3. The arithmetic mean and SEM were calculated. The results on the effect of marinating meat with buttermilk and sour milk were verified using a one-way analysis of variance. Significant differences between the means in groups were estimated by Duncan’s test. Differences were considered significant if *p* < 0.05. The results on the effect of marinating on the sensory properties of roast products were verified with the use of non-parametric Kruskal–Wallis tests.

## 3. Results and Discussion

The study showed that the breast muscles of hens marinated in sour milk showed greater absorption of the marinade than in buttermilk (*p* < 0.05) (Table 1). The absorption of the marinade had a beneficial effect on the quality of the meat. According to Yosop [13], the use of sour marinades in the marinating process is an effective method of improving the technological and functional properties of meat. Mozuriene et al. [18] found a beneficial effect of acidic marinades on the water-holding capacity of meat, which in turn is associated with the swelling of myofibrillar proteins as well as an increase in ionic strength and a decrease in pH [19,20,21].

In the present study, the acidity of raw breast muscles of hens marinated with buttermilk and sour milk was lower (*p* < 0.05) than the pH of non-marinated breast muscles (Table 1), which can be explained by the effect of fermented milk products used in the marinating process, the pH of which was 5.53 for buttermilk and 5.35 for sour milk. The effect of the marinade pH on the meat pH after marinating was also reported by Kim [22], Kumar et al. [23], Wójciak et al. [24], and Latoch and Libera [25]. Additionally, after heat treatment (roasting), the pH of marinated breast muscles was lower than that of non-marinated ones (Table 1). The study by Kumar et al. [23] showed that the decrease in pH resulting from acid marinating had a positive effect on texture, increasing the water absorption of hen meat after laying. Meat pH values can also affect the physicochemical properties of meat, including colour, WHC, and tenderness [26].

The obtained results indicate an increase (*p* < 0.05) in the water absorption of marinated products, both with sour milk and buttermilk (Table 1). Additionally, Gault [27] found that meat marinated with the immersion method in sour marinades had a pH below 5.0, absorbed water better, had less cooking loss, and was less hard compared to the control. 

Colour is an important indicator of the technological quality of meat and meat products [21,28]. In the present study, it was found that after marinating with buttermilk, the meat from the breast muscles was characterised by higher brightness (L*), similar redness (a*), and lower yellowness (b*) on the surface and cross-section compared with non-marinated meat (Table 1), which proves the influence of the marinating process with fermented milk products used on the colour of raw meat from the breast muscles. According to Fernandez-Lopez et al. [29] and Hui [30], the colour of meat is mainly related to the content of a specific haem dye, the chemical state of the dyes, and the scattering and light-absorbing properties. The lighter colour (higher values of the L* parameter) of the marinated meat in the present study probably resulted from the lower pH value of the meat after marinating. Swatland [31] found that the brightness of meat is closely related to the pH value. Low pH leads to increased light scattering and high L* values. An increase in the value of the L* brightness parameter was also noted by Wójciak et al. [32] by marinating the maturing beef with whey. However, Latoch [28] did not find an effect of marinating pork loin with buttermilk, kefir, or yoghurt on the value of the L* parameter. Strzyżewski et al. [33] reported that a change in the acidity of meat may cause changes in the L* and b* parameters. According to Latoch et al. [21], changes in the degree of the red colour saturation reflect the processes taking place in the process of marinating meat. 

The analysis of the Warner–Bratzler maximum shear force results showed (*p* < 0.05) a change in the mechanical properties of marinated products, both raw and heat-treated, compared to non-marinated meat (Table 2). Products marinated in buttermilk and sour milk were characterised by a lower shear force F (max) compared to the control group (NM). Numerous studies [8,20,21,25,34] show that the use of sour marinades has a direct impact on the texture characteristics of meat and meat products. The studies by Ergezer and Gokce [34] showed a beneficial effect of the addition of lactic acid when marinating turkey meat on the hardness measured by the shear force. 

The present author’s study, based on the measurement of deformations occurring during the compression of the samples, showed that the use of buttermilk and sour milk had a positive effect on the reduction in hardness and secondary texture parameters related to this trait, i.e., the cohesiveness and chewiness of raw and roasted breast muscles of RIR hens (*p* < 0.05) compared to non-marinated muscles from the control. Additionally, studies by Latoch et al. [21] and Latoch [28] showed that the use of fermented milk products, kefir, yoghurt, and buttermilk, for marinating pork reduced the hardness and chewiness of pork loin and sous-vide steaks. An increase in meat tenderness was correlated with an increase in water-holding capacity and increased extraction of myofibrillar proteins. These changes can be explained by physicochemical mechanisms resulting mainly from a decrease in pH and an increase in ionic strength [8,20]. The positive effect of marinating with kefir on the hardness and springiness of wild boar meat was noted by Żochowska and Kujawska et al. [8]. 

The optimal microbiological quality of the marinated meat product occupies a special position in affecting its safety. Mesophilic aerobic bacteria and bacteria of *Pseudomonas* spp. are common in poultry meat and their level is an indicator of meat freshness. The present study (Table 3) showed that the total number of mesophilic aerobic microorganisms and bacteria of *Pseudomonas* spp. in the meat of RIR hens before marinating was 3.92 log cfu/g and 4.26 log cfu/g, respectively, which indicates that the raw material met the normative requirements of microbiological quality. One of the methods of limiting the multiplication of unfavourable microflora may be the use of acid marinades with the use of fermented milk products containing lactic acid bacteria strains, which was confirmed by our research. Both the use of buttermilk and sour milk as a marinade reduced the increase in the number of mesophilic aerobic bacteria and *Pseudomonas* spp. in marinated raw and roasted products (Table 3). All marinated products contained acceptable threshold values for these microorganisms (less than 10 log cfu/g or not at all). The study by Latoch and Libera [25] showed that marinating pork in buttermilk and yoghurt increased the safety of cooked steaks by reducing the number of mesophilic and psychrotrophic aerobic bacteria. Confirmation of the beneficial effect of marinating with buttermilk and whey on limiting the growth of mesophilic aerobic bacteria and *Pseudomonas* spp. in raw and roasted breast muscles pheasants was also noted by Augustyńska-Prejsnar et al. [35]. Bacteria from the Enterobacteriaceae family are associated with the spoilage of poultry meat under conditions conducive to their growth [6]. Enterobacteriaceae representatives (*Citrobacter*, *Enterobacter*, *Hafnia*, *Klebsiella*, *Providencia*, *E. coli*, *Yersinia*, *Proteus*) were identified in meat and poultry products [4,36,37,38]. In the present study, 3.12 log cfu/g of Enterobacteriaceae were found in the non-marinated raw meat of RIR hens. In raw meat marinated in sour milk, the level of Enterobacteriaceae remained at a similar level (3.10 log cfu/g), while the marinating process using buttermilk significantly (*p* < 0.05) reduced the number of Enterobacteriaceae colony-forming units in raw meat to 2.63 log cfu/g. After roasting, the presence of Enterobacteriaceae was found only in the control group (non-marinated) in the amount of 1.64 log cfu/g. As the literature indicates [38,39,40], lactic acid bacteria cultures produce numerous substances with antimicrobial activity, such as, for example, organic acids and bacteriocins [38], including bacteriocins that inhibit the growth of Enterobacteriaceae [39]. The presence of lactic acid bacteria limited the development of saprophytic and pathogenic bacteria in the raw maturing meat product. The reduction in Enterobacteriaceae in dry sausages fermented without the addition of nitrites was described by Kononiuk and Karwowska [40] after the use of acid whey. 

**Figure 1:**
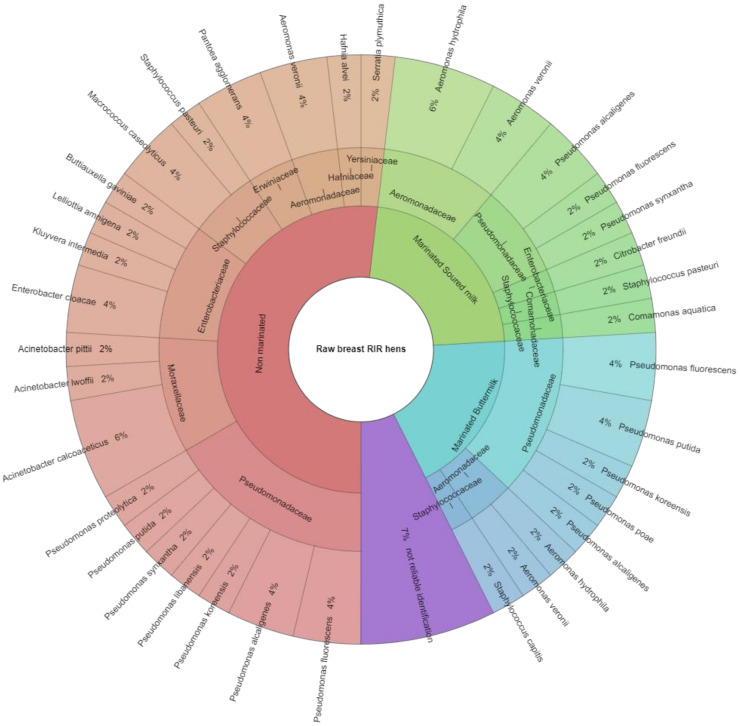
Bacteria identified in samples of raw meat from RIR hen breast muscles.

**Figure 2:**
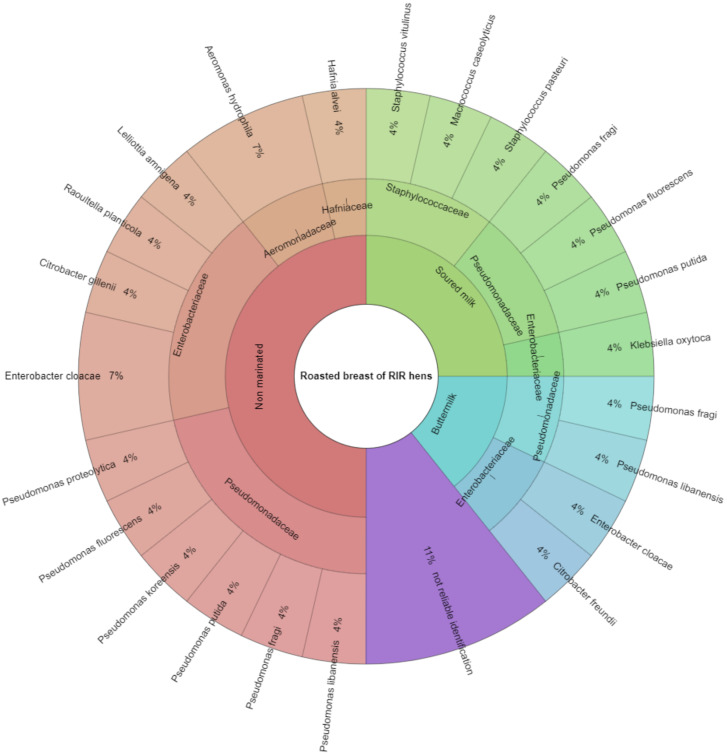
Bacteria identified in samples of roasted meat from RIR hen breast muscles.

The results of the identification of microorganisms in raw and heat-treated (roasted) hen meat from the breasts of RIR hens using a MALDI-TOF MS Biotyper are shown in Figure 1 and Figure 2. The figures take into account the results with a point value of ≥2.00 for both the species and the type of bacteria. Raw meat samples were evaluated in three groups, i.e., in the control group, which consisted of non-marinated muscles (NM), in the group of muscles marinated in buttermilk (BM), and in the group of muscles marinated in sour milk (SM). Identification was made for 82 samples of microorganisms isolated from RIR hen meat, of which about 93% were fully identified. Bacteria isolated from 55 samples from raw meat were unambiguously assigned to 10 bacterial families and 26 strains. In 27 samples isolated from roasted meat, 5 bacterial families and 16 strains were identified. The obtained results indicate that the process of marinating with fermented milk products reduced the number of identified mesophilic aerobic families and bacteria, both in raw and roasted meat samples. In samples obtained from raw hen meat, eight families were isolated from the control group (NM), including Pseudomonadaceae, represented by *Pseudomonas fluorescens* (4%) and *Pseudomonas alcaligenes* at 4%, and *Pseudomonas koreensis*, *Pseudomonas libanensis*, *Pseudomonas synxantha*, *Pseudomonas putida*, and *Pseudomonas proteolytica* at 2% each. The most frequently isolated bacterial strain of the Moraxellaceae family was *Acinetobacter calcoaceticus* (6%); of the Enterbacteriaceae family, *Enterobacter cloacae* were isolated (4%), as well as *Kluyvera intermedia*, *Lelliottia amnigena*, and *Buttiauxella gaviniae*, 2% each. Of the Staphylococcaceae family, *Macrococcus caseolyticus* (4%) and *Staphylococcus pasteuri* (2%) were isolated. Of the Erwiniaceae family, 4% *Pantoea agglomerans* were isolated; of the Aeromonadaceae family, *Aeromonas veronii*; of the *Yersiniaceae* genus, *Serratia plymuthica*; and of the *Hafniaceae* genus, *Hafnia alvei*. Seven percent of bacterial isolates were unidentified. 

In the group of raw muscles marinated with buttermilk, three families were isolated, i.e., Pseudomonadaceae, of which the most frequently isolated strains were *Pseudomonas fluorescens* and *Pseudomonas putida* (4% each); as well as Aeromonadaceae and Staphylococcaceae, of which single species of bacteria have been isolated. In raw muscles marinated with sour milk, representatives of five families were identified, i.e., Pseudomonadaceae represented mainly by *Pseudomonas alcaligenes* (4%); Aeromonadaceae, of which *Aeromonas hydrophila* (6%) and *Aeromonas veronii* (4%) were most frequently isolated; and Enterobacteriaceae, Staphylococcaceae, and Comamonadaceae, of which individual bacterial species were isolated. Additionally, after heat treatment (roasting), the number of identified families in the group of muscles marinated with buttermilk was lower by half than in the group of non-marinated muscles. In the control group (non-marinated muscles), four families were identified (Pseudomonadaceae, Enterobacteriaceae, Aeromonadaceae, Hafniaceae), in meat marinated with buttermilk, there were two families (Enterobacteriaceae, Pseudomonadaceae), and in meat marinated with sour milk, three families (Pseudomonadaceae, Enterobacteriaceae, Staphylococcaceae). Of bacterial isolates, 11% were unidentified (point value < 2.00). 

The presented results of the microbiological assessment indicate that marinating with buttermilk and sour milk resulted in an improvement in the microbiological quality of hen meat. 

The presence of pathogenic bacteria (*Salmonella* spp.) was not found in raw or marinated chicken meat, which proves the good health condition of the hens from which the meat was obtained, good sanitary conditions during the use and slaughter of laying hens, and proper conditions during the storage of the meat. 

The sensory analysis showed (Table 4) that marinating meat from the breast muscles of hens after the first year of laying with buttermilk (MB) and sour milk (MS) has a beneficial effect on the tenderness, colour, and juiciness of roasted hen breast meat compared to the control (NM). The obtained results are consistent with the results of the study by [36], who found that the sensory characteristics of poultry food depend on the raw meat quality and the processing method used, including marinating. Additionally, the study by Kumor et al. [23] showed that marinades with organic acids can be used to improve the tenderness and juiciness of hen meat after laying. Vlahova-Vangelova et al. [41] demonstrated a beneficial effect of whey marinating on the tenderness of broiler chicken meat after heat treatment with the grilling method. The results of the effect of acid marinades on the sensory quality of meat of other animal species are also known. Kim [22] found that the use of sour whey to marinate beef improved its tenderness and juiciness compared to the control group. In the study by Żochowska-Kujawska et al. [8], the use of kefir for marinating improved the tenderness, juiciness, and overall attractiveness of wild boar meat. 

## 4. Conclusions

It was shown that the use of buttermilk and sour milk for marinating the meat of RIR hens after one year of laying use had an impact on the quality characteristics of the meat. Marinating meat with fermented dairy products lightened the colour, lowered the shear force, reduced hardness and chewiness, and limited the growth of aerobic bacteria and *Pseudomonas* spp. Eight bacterial families were identified in the samples of raw non-marinated meat, three families in the group of raw muscles marinated with sour milk, and five in the group of raw muscles marinated with sour milk. Additionally, after heat treatment, the number of identified families in the group of muscles marinated with buttermilk and sour milk was smaller than in the group of non-marinated muscles. The sensory evaluation showed a beneficial effect of the fermented milk products used on the tenderness, juiciness, and colour of roasted meat. The obtained results allow us to conclude that marinating with fermented milk products creates new opportunities and prospects for the culinary use of the meat of RIR hens after one year of laying use. The use of buttermilk and sour milk as a marinade for the meat of hens after one year of laying use, in the conditions of free-range breeding, allows obtaining a product of high microbiological quality, good technological characteristics, and high sensory acceptability. 

## Figures and Tables

**Table 1:** Effect of marinating with fermented milk products on the physical characteristics of raw and roasted hen breast meat.

Parameter	Raw Breast of RIR Hens				Roasted Breast of RIR Hens			
	NM ^1^	MB ^2^	MS ^3^	SEM	NM ^1^	MB ^2^	MS ^3^	SEM
Marinade absorption (%)	-	8.96 a ± 0.85	11.15 b ± 0.92	0.44	-	-	-	-
pH	5.70 a ± 0.01	5.53 b ± 0.02	5.35 c ± 0.01	0.03	6.01 a ± 0.02	5.72 b ± 0.01	5.73 b ± 0.03	0.03
Water holding capacity (%)	28.11 a ± 2.12	38.68 b ± 3.06	39.55 b ± 3.50	1.02	-	-	-	-
Colour (cross-section)								
Lightness (L*)	53.63 a ± 3.62	58.89 b ± 3.80	59.59 b ± 4.12	0.50	79.79 a ± 4.03	85.68 b ± 3.75	85.44 a ± 4.15	0.79
Redness (a*)	1.54 ± 0.20	1.30 ± 0.35	1.32 ± 0.38	0.06	2.11 ± 0.40	1.98 ± 0.32	2.09 ± 0.51	0.12
Yellowness (b*)	5.36 a ± 0.90	4.10 b ± 0.82	4.13 c ± 0.86	0.16	10.81 ± 1.12	11.18 ± 0.90	11.30 ± 0.89	0.18
Colour (surface)								
Lightness (L*)	53.08 a ± 3.50	66.89 b ± 4.12	74.11 c ± 3.34	1.24	78.06 a ± 4.60	79.53 a ± 4.15	75.39 b ± 3.40	1.24
Redness (a*)	1.25 ± 0.21	1.23 ± 0.62	1.16 ± 0.40	0.11	1.81 a ± 0.35	1,30 b ± 0.40	1.77 a ± 0.48	0.11
Yellowness (b*)	3.73 a ± 0.60	2.78 b ± 0.72	2.63 b ± 0.63	0.11	14.98 a ± 1.15	10.85 b ± 1.03	11.25 b ± 2.13	0.11

^1^ NM—non-marinated control group; ^2^ MB—marinated in buttermilk; ^3^ MS—marinated in sour milk; a, b, c—values in rows with different letters differ significantly, *p* < 0.05.

**Table 2:** Effect of marinating with fermented milk products on texture parameters (Warner–Bratzler, texture profile analysis) of raw and roasted hen breast muscles.

Parameter	Raw Breast of RIR Hens				Roasted Breast of RIR Hens			
	NM ^1^	MB ^2^	MS ^3^	SEM	NM ^1^	MB ^2^	MS ^3^	SEM
Warner–Bratzler shear force (N)	31.92 a ± 3.12	28.83 b ± 2.56	29.30 b ± 3.04	0.32	29.41 a ± 1.98	25.58 b ± 2.89	27.33 b ± 2.54	0.47
Texture profile analysis (TPA)								
Hardness (N)	26.50 a ± 3.92	23.20 b ± 2.80	21.93 b ± 2.14	0.76	25.89 a ± 2.94	21.72 b ± 3.20	20.24 b ± 1.96	1.82
Resilience	0.29 ± 0.06	0.27 ± 0.05	0.26 ± 0.04	0.01	0.20 ± 0.06	0.21 ± 0.05	0.25 ± 0.03	0.02
Cohesiveness	0.39 a ± 0.08	0.24 b ± 0.04	0.30 a ± 0.06	0.02	0.41 a ± 0.08	0.23 b ± 0.03	0.24 b ± 0.04	0.10
Springiness (mm)	1.97 ± 0.30	2.22 ± 0.40	2.18 ± 0.28	0.05	2.78 ± 0.32	2.46 ± 0.38	2.63 ± 0.50	0.46
Chewiness (N)	20.40 a ± 2.30	13.08 b ± 1.85	15.36 b ± 2.15	0.04	30.60 a ± 3.12	14.06 b ± 2.30	13.30 b ± 2.84	2.92

^1^ NM—non-marinated control group; ^2^ MB—marinated in buttermilk; ^3^ MS—marinated in sour milk; a, b—values in rows with different letters differ significantly, *p* < 0.05.

**Table 3:** Effect of marinating with fermented milk products on the microbiological parameters of raw and roasted hen breast muscles.

Parameter	Raw Breast of RIR Hens				Roasted Breast of RIR Hens			
	NM ^1^	MB ^2^	MS ^3^	SEM	NM ^1^	MB ^2^	MS ^3^	SEM
Mesophilic aerobic bacteria (log cfu/g)	3.92 a ± 0.18	2.78 b ± 0.15	2.94 b ± 0.32	0.06	2.86 a ± 0.24	1.84 b ± 0.36	1.98 b ± 0.47	0.04
*Pseudomonas* spp. (log cfu/g)	4.26 a ± 0.12	2.40 b ± 0.18	2.35 b ± 0.20	0.09	3.04 a ± 0.18	1.12 b ± 0.28	1.68 b ± 0.40	0.08
Enterobacteriaceae (log cfu/g)	3.12 a ± 0.10	2.63 b ± 0.08	3.10 a ± 0.27	0.08	1.64 ± 0.14	nd	nd	nd

^1^ NM—non-marinated control group; ^2^ MB—marinated in buttermilk; ^3^ MS—marinated in sour milk; a, b—values in rows with different letters differ significantly, *p* < 0.05.

**Table 4:** Sensory traits of roasted breast meat, marinated and non-marinated with fermented milk products.

Parameter	Roasted Breast of RIR Hens			
	NM ^1^	MB ^2^	MS ^3^	SEM
Odour intensity	3.92 ± 0.36	4.14 ± 0.52	3.86 ± 0.40	0.06
Odour desirability	4.57 ± 0.46	4.50 ± 0.61	4.42 ± 0.35	0.05
Flavour intensity	4.71 ± 0.68	4.77 ± 0.52	4.64 ± 0.42	0.08
Flavour desirability	4.29 ± 0.42	4.38 ± 0.50	4.29 ± 0.64	0.08
Juiciness	4.07 a ± 0.46	4.57 b ± 0.36	4.63 b ± 0.43	0.06
Tenderness	3.79 a ± 0.35	4.42 b ± 0.40	4.38 b ± 0.63	0.09
Cross-section colour	4.21 a ± 0.52	4.64 b ± 0.40	4.57 b ± 0.48	0.06
General appearance	4.57 ± 0.56	4.64 ± 0.63	4.57 ± 0.44	0.05

^1^ NM—non-marinated control group; ^2^ MB—marinated in buttermilk; ^3^ MS—marinated in sour milk; a, b—values in rows with different letters differ significantly, *p* < 0.05. Explanation of the scale sensors used for the evaluation: odour, flavour intensity: 1—changed, 2—moderately changed, 3—typical, weak; 4—typical, strong, 5—typical, very strong; odour, flavour desirability: 1—not desirable, 2—fairly desirable, 3—desirable, 4—very desirable, 5—highly desirable; juiciness: 1—very dry, 2—dry, 3—slightly juicy, 4—juicy, 5—very juicy; tenderness: 1—very hard, 2—hard, 3—slightly tender, 4—tender, 5—very tender.

## Data Availability

Data are available from the corresponding author.
